# New Method of Treating a Professional Rival

**Published:** 1847-06

**Authors:** 


*New Method of Treating a Professional Rival.*—We strongly recommend the following to gentlemen who are not over scrupulous in their integrity towards those who cross their paths, or presume to dispute their professional supremacy. It is not, perhaps, quite so safe as the knowing nod and wink plan, or the solemn and confidential communication of plausible folk, but it is more off-hand, and employed with *due* caution may be made available to the members of the dental profession as well as the medical.

"The case is reported in the *Gazette Medicale*, to have occurred recently at Bethune in France. A medical man newly arrived in a neighborhood where there was already a highly-esteemed practitioner in good practice, commenced his career by endeavoring to injure the reputation of this gentleman, resorting to the basest calumnies in order to attain his object. Finding that his efforts to bring M. Bacqueville, the practitioner referred to, into disrepute, had completely failed, he at length spread a report that a lady of high rank had been clandestinely delivered by this gentleman, and that the child had been made away with! When this report was fairly in circulalation, the calumniator proceeded to a cemetery, disinterred a child, and threw it into a privy on the premises of M. Bacqueville; he then addressed a letter to the authorities, accusing M. Bacqueville of having murdered a child, and pointed out the place where the body would be found. Accordingly the premises of M. Bacqueville were searched, the body of a child discovered, and that gentleman was taken to prison, where he remained confined for a considerable time. He was at length liberated on account of there being no evidence against him. From some cause or other, shortly after this, the scandalous proceeding was discovered, and public indignation being roused, the man was brought before the authorities, and charged with an offence *contra bonos mores*, in disinterring a body. He was found guilty, and sentenced to a year's imprisonment, and a fine of 3000 francs (£120.)"

